# Antenna Modeling and Reconstruction Accuracy of Time Domain-Based Image Reconstruction in Microwave Tomography

**DOI:** 10.1155/2013/343180

**Published:** 2013-03-28

**Authors:** Andreas Fhager, Shantanu K. Padhi, Mikael Persson, John Howard

**Affiliations:** ^1^Biomedical Engineering Division, Department of Signal and Systems, Chalmers University of Technology, 41296 Gothenburg, Sweden; ^2^Curtin Institute of Radio Astronomy (CIRA), ICRAR, Curtin University, Perth, WA 6102, Australia; ^3^PRL, Research School of Physics and Engineering, Australian National University, Canberra, ACT 0200, Australia

## Abstract

Nonlinear microwave imaging heavily relies on an accurate numerical electromagnetic model of the antenna system. The model is used to simulate scattering data that is compared to its measured counterpart in order to reconstruct the image. In this paper an antenna system immersed in water is used to image different canonical objects in order to investigate the implication of modeling errors on the final reconstruction using a time domain-based iterative inverse reconstruction algorithm and three-dimensional FDTD modeling. With the test objects immersed in a background of air and tap water, respectively, we have studied the impact of antenna modeling errors, errors in the modeling of the background media, and made a comparison with a two-dimensional version of the algorithm. In conclusion even small modeling errors in the antennas can significantly alter the reconstructed image. Since the image reconstruction procedure is highly nonlinear general conclusions are very difficult to make. In our case it means that with the antenna system immersed in water and using our present FDTD-based electromagnetic model the imaging results are improved if refraining from modeling the water-wall-air interface and instead just use a homogeneous background of water in the model.

## 1. Introduction

Microwave imaging has received significant attention in the research community during the last couple of decades as a modality that potentially could improve the diagnostics of, for example, breast cancer tumors. Recent progress in the field has been reviewed in [1] and [2]. Today the research has come to the stage where early clinical trials have been and are being performed, [3–6]. The results from the clinical work are promising, but further development of the measurement systems as well as of the image reconstruction algorithms remains before the technique can be considered for daily clinical practice.

When performing microwave tomography the aim is to quantitatively reconstruct the dielectric parameters in the region under test. This involves solving a computationally challenging nonlinear and ill-posed optimization problem. The image reconstruction algorithm utilizes measured data that are compared against a corresponding numerical simulation of the system, and the dielectric profile is iteratively updated based on the difference between the simulation and the measurement. Even though this comparison requires a realistic numerical model for the best accuracy, most of the published works have used 2D models together with a calibration procedure to enable the comparison with experimental data. Largely, this can be attributed to a significant increase in the computational load when moving from 2D to three-dimensional (3D) modeling. However the electromagnetic scattering and consequently the reconstruction problems are inherently a 3D problem. Furthermore it is usually not possible to create realistic antenna models in 2D, except for line source antennas. By using a 2D model to solve the inverse scattering problem inaccuracies will therefore inevitably be introduced in the reconstructed image. This problem has been identified by the research community, and, with the ever increasing computational resources available these days, the focus is now more and more turning to solving the full 3D problem. Recent works using 3D algorithm have been reported in [7–11].

In this paper we show several examples where images have been reconstructed from scattering data in order to discuss and illustrate the need for accurate modeling of the antenna system and its geometry to enable robust image reconstruction. The aim is also to get an understanding of what accuracy we could realistically expect in the reconstruction and how it is affected by various modeling errors. Examples of targets placed in a surrounding of air are studied, and in an effort to approach more biologically relevant settings we have studied examples where the antenna system was entirely immersed in water. The examples studied in this paper are entirely based on experimental measurement data. Our reconstruction algorithm, described in [12], is based on FDTD modeling to solve the forward scattering problem and the adjoint Maxwell's equations to compute gradients used in an iterative optimization procedure.

The paper is organized as follows. In Section 2, the theoretical background of our work is described including FDTD methods with the minimization procedure. The experimental prototype is described in Section 3. In Section 4, the forward modeling is investigated, and the corresponding imaging results originating from experimental data are presented and discussed. And finally the conclusions are drawn in Section 5.

## 2. Theoretical Background

An iterative electromagnetic time-domain inversion algorithm has been used and applied in estimating the dielectric parameters of different test objects. The foundation of the algorithm is an electromagnetic solver based on the FDTD method, [13], is used to numerically model the antennas and to simulate the field propagation inside the system. The same solver is used to compute the adjoint Maxwell problem which is required for the gradient computation in the optimization algorithm. The adjoint field is used extensively elsewhere in various types of inverse problems see, for example, [14, 15]. The algoithm used here is described in [12]. A correspoding 2D version has also been described in detail in [16, 17].

Our basic idea for solving the inverse electromagnetic problem is to use scattering measurements of wideband pulses for several transmitter/receiver combinations surrounding a region of interest and thereafter to compare the measured data in the time domain with a corresponding numerical simulation of the system. In the first iteration one starts with comparing the measurement with a simulation of an empty antenna system. Thus there is a difference between the measured and the simulated data, and this difference is used to update the dielectric properties inside the region of interest. In this way the dielectric distribution is iteratively refined until the desired agreement between the simulated and measured signals has been achieved. The underlying assumption for this approach is that as the difference between the simulated and measured data is decreasing, the reconstruction is also converging. In other words, the aim of the reconstruction procedure is to minimize the objective functional, *F*, defined as

(1) $$\begin{array}{c} F\left(\epsilon ,\sigma \right)=\int_{0}^{T}\sum_{m=1}^{M}\;\sum_{n=1}^{N}{\left|E_{mn}\left(\epsilon ,\sigma ,t\right)-E_{mn}^{m}\left(t\right)\right|}^{2}dt, \end{array}$$

where **E**_*mn*_(*ϵ*, *σ*, *t*) is the calculated field from the computational model and **E**_*mn*_^*m*^(*t*) is the corresponding measured data when antenna number *m* has been used as transmitter and antenna *n* as receiver. *M* is the number of transmitters, *N* is the number of receivers, and *T* is the duration of the pulse. In a 2D TM-mode formulation only one spatial field component is used in (1), but for the 3D formulation it is necessary to include all three spatial components.

In search for the minimum of the objective functional it is differentiated with respect to the dielectric components by a first order perturbation analysis. In this way the Fréchet derivatives with respect to the conductivity and the permittivity of the functional are used to define gradients, in every grid point, inside the region. The gradients are used with the conjugate-gradient method together with the successive parabolic interpolation line search to minimize the objective functional. The reconstruction procedure is then iterated with the objective functional as a measure to monitor the convergence and to determine when the reconstruction is completed. Usually the minimization procedure converges within 10–20 iterations.

In the 2D simulations it is not possible to construct a realistic antenna model, but the transmitter is only modeled with a hard point source, in which the field strength is prescribed at the source position. At the receiver locations the field values are sampled directly from the corresponding E-field component in the grid. By contrast, the 3D algorithm allows for realistic antenna models. We are using the thin-wire approximation to model the monopoles, [18], and the RVS with 50 *Ω* impedance to model the feed at the transmitting, receiving, and inactive antennas, [19, 20]. Furthermore the ground plane in our experimental system is modeled as a perfect electric conductor; that is, the corresponding field components in the FDTD grid are set to zero. The walls of the tank have dielectric properties close to those of air and are therefore neglected in the numerical model. Outside the antenna system the computational grid is truncated by the CPML absorbing boundary condition implying that the region outside the antenna array is treated as open empty space. In the case where the tank is filled with a liquid it has been modeled as a cubic volume with side lengths equal to the inner measures of the tank and a height equal to the level of the liquid. Even in this case we have not modeled the tank material but instead treated everything outside as empty space and terminated the computation domain with CPML. A more detailed discussion and validation of the FDTD antenna array model with a comparison against experimental data measured with the antennas in air can be found in our previous work [21].

As already mentioned the solution of the inverse problem heavily relies on the comparison between the measured and the simulated scattering data. To compensate for systematic modeling errors a calibration procedure of the measured data is used such that

(2) $$\begin{array}{c} E_{\text{cal}}^{m}\left(f\right)=\frac{S_{\text{scat}}^{m}\left(f\right)}{S_{\text{ref}}^{m}\left(f\right)}\;E_{\text{ref}}^{s}\left(f\right). \end{array}$$

*S*_scat_^*m*^ is the measured reflection and transmission coefficients of the test object, *S*_ref_^*m*^(*f*) is a reference measurement of an empty system, and *E*_ref_^*s*^(*f*) is a corresponding reference simulation. Finally *E*_cal_^*m*^(*f*) is the calibrated data used for comparing the FDTD simulations in the reconstruction process. This calibration procedure has been applied to the measured data for both the 2D and the 3D imaging examples in this paper.

In our experimental prototype the antennas are positioned in a plane. The possibility to accurately reconstruct out-of-plane objects is thus very limited: to do so it would be necessary also to make additional measurements outside the antenna plane. To allow imaging with the 3D algorithm of a test object with finite height, we implemented a heuristic pseudo-3D technique that assumed constant properties of the test object as a function of height, *z*, above the ground plane. The gradients computed in the grid cell plane immediately above the ground plane were copied upwards to the height of the test object. This method needs a priori information about the height of the reconstructed target. Since the reconstruction problem is both nonlinear and ill-posed, the resulting image strongly depends on the adopted regularization technique, the initialization of the reconstruction, and also the spectral content of the pulse. Here we used the same techniques as described in [12] to overcome these challenges.

## 3. Experimental Setup

**Figure 1:**
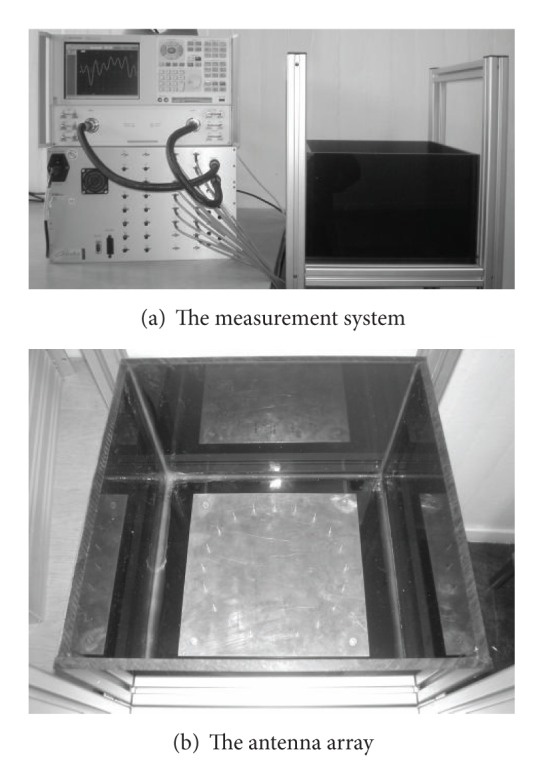
(a) The system consists of a VNA, a switch, and an antenna array. (b) Closeup of the antenna array placed inside a tank. The monopoles are seen mounted in a circle over the ground plane. The entire antenna system is mounted inside a tank made of perspex sheets.

The measurement strategy is to measure the multistatic scattering matrix at a large number of frequencies and to use that data to generate a time-domain pulse via an inverse Fourier transformation. In the experimental system 20 monopole antennas, each of length 19.5 mm and diameter 0.8 mm, are arranged evenly distributed on a circle with radius 100 mm. The circle of antennas is centered on a square ground plane with side length 250 mm mounted at the bottom of a tank, made of 1 cm thick perspex sheets with inner measures 350 × 350 mm^2^. To measure the multistatic matrix each antenna is operated as a transmitter as well as a receiver. The microwave measurements are made with network analyzer Agilent E8362 B PNA which is a two-port network analyzer. To fully control the experiment a 2:32 switch multiplexer module, Cytec CXM/128-S-W, is used to automatically connect and disconnect the different combinations of antenna pairs to the network analyzer.  Figure 1 shows a photograph of the antenna array.

## 4. Results and Discussion

In our previous publication, [21], a detailed study was made of the FDTD modeling compared to measured data of an empty system, and the accuracy of the modeling was verified. One of the aims of the present paper is to study how errors in the antenna model, for example, the monopole length, of the FDTD model impact the reconstruction. To do so we study the antenna modeling and image reconstruction both when the tank is empty and when it is filled with water. The reason why it is interesting to study the antenna system immersed in water is that water is a good model for the matching medium that has to be used when applying microwave tomography to imaging the interior of the human body. Without a matching medium the majority of the irradiated energy would be reflected from the skin, thereby never penetrating into the body and producing useful data.

To enable a quantitative evaluation of the accuracy of the reconstructed images we have adopted the relative squared error of the image, and for the permittivity image it is defined in (3), and analogously for the conductivity image. The integration of the relative squared error is made over the reconstruction domain *Ω* where *r* < *R*_*rd*⁡_,

(3) $$\begin{array}{c} \delta =\frac{\int_{\Omega }{\left|\epsilon_{\text{original}}-\epsilon_{\text{reconstructed}}\right|}^{2}dS}{\int_{\Omega }{\left|\epsilon_{\text{original}}-\epsilon_{\text{background}}\right|}^{2}dS}. \end{array}$$

### 4.1. Reconstruction of a Single Dielectric Target in Air

**Figure 2:**
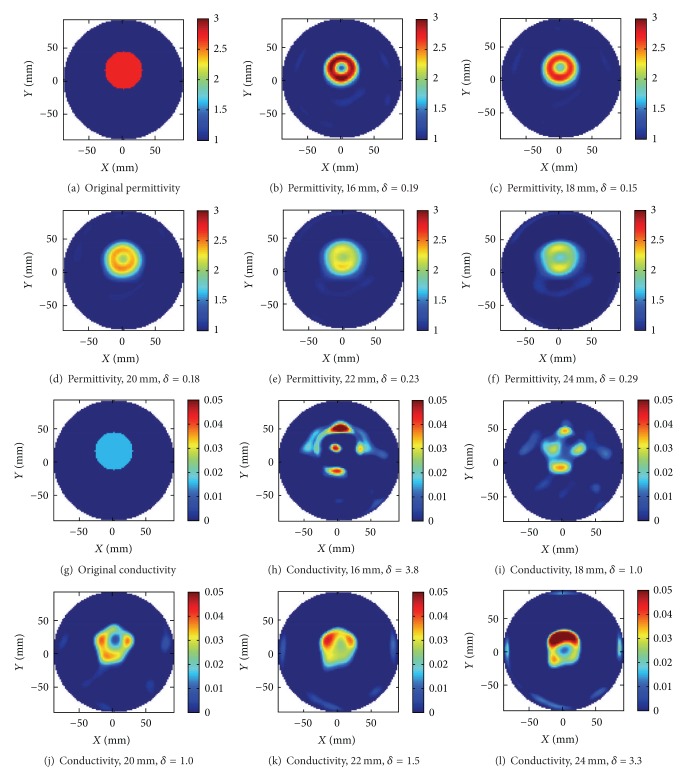
Reconstructed images of the sunflower oil target using monopole lengths 16, 18, 20, 22, and 24 mm, respectively. Both permittivity and conductivity images are shown, and in (a) and (g) the original dielectric distribution has been illustrated. The original target had *ϵ*_*r*_ = 2.7 and *σ* = 0.015  S/m at 2.3 GHz and had diameter 56 mm.

With the purpose to study how the reconstructed image is affected by errors in the length of the monopole antenna model, we first studied a single dielectric target in an otherwise empty antenna array. The imaging situation was the same as in our previous publication, [12], that is, a single target made of sunflower oil surrounded by air. The dielectric properties of the target were *ϵ*_*r*_ = 2.7 and *σ* = 0.015 S/m at 2.3 GHz. It was shaped like a cylinder with diameter 56 mm and height 20 mm and was placed at 14 mm offset in the *y* direction from the center of the antenna array. It had constant properties along its height in the *z* direction, and thus it is only necessary to show a cross-sectional slice of the dielectric profile. The FDTD model data is summarized in Table 1. A cylindrical volume of height 20 mm and radius *R*_*rd*⁡_ = 90 mm centered in the antenna array was used as the reconstruction domain together with the pseudo-3D approach described earlier. To investigate the accuracy of the reconstructed image with respect to the modeling, different lengths of the monopoles were used in the numerical electromagnetic model. The length of the monopoles in the antenna system is 19.5 mm, but images were reconstructed modeling the length as 16, 18, 20, 22, and 24 mm, respectively. This resulted in a change in the computed resonance frequency and the associated signal strength. The reconstruction results together with an illustration of the original dielectric distribution are shown in Figure 2. The results obtained with 20 mm monopole length in Figures 2(d) and 2(j) are identical to what was published earlier [12] and represent a scenario where the numerical modeling at the given grid size is as close as possible to the experimental system. As a measure of reconstruction accuracy the relative error as defined in (3) has been calculated for each reconstructed image, and it is shown in the figure below the respective reconstruction. The reconstructed object permittivities are on average about *ϵ*_*r*_ = 2.1, 2.2, 2.4, 2.7, 3.0, respectively, for the various monopole lengths. The minimum value is 12.5% smaller and the maximum value is 25% larger than what was obtained for the 20 mm monopole length. The recontructions of the conductivity are, however, not accurate, but it is clearly evident that the artifacts increase the more the monopole lengths deviate from the real value. The reason why the reconstruction is less accurate is that the difference in the imaginary part of the complex dielectric permittivity between the object and background is only a fraction in comparison with the difference in the real part. Reconstruction errors in the real part therefore overwhelm attempts to reconstruct the imaginary part, and consequently the accuracy is reduced. Compared to the original dielectric profile, however, the most accurate reconstruction of the permittivity is obtained for the 18 mm monopole. This reflects the deviaton of half a grid cell between the 20 mm antenna model and the precise length of the monopole with the real length being 19.5 mm. But also there is a tolerance in the cutting of the monopoles of about 0.5 mm. Numerical uncertainties in the FDTD solution and errors in the dielectric measurement of the sunflower oil are other reasons.

**Figure 3:**
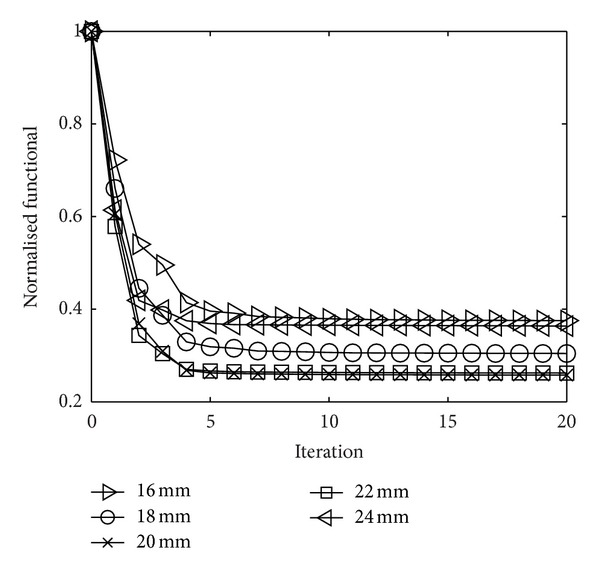
Normalized functional values as a function of the iteration number for the reconstructions of the sunflower target.

In Figure 3 the functional values of the reconstructions have been plotted. In all cases the starting values have been normalized to one. Firstly these plots illustrate the convergence of the reconstruction process, but it also shows that the lowest functional value was obtained for the reconstruction with the 20 mm monopole. A nice illustration of the ill-posedness of the problem is that the difference between the 20 mm and the 22 mm case is on the verge of being negligible even if the difference in the reconstructed image is certainly not. Another conclusion from this result is that a model error in the antenna length of only one grid cell resulted in a considerable change in the reconstructed image. In summary these results clearly show the need of using an accurate antenna length in order to maximize the accuracy of the reconstructed image.

### 4.2. Evaluation of the Forward Simulation with the Antenna Array Immersed in Water

In this section we study the situation when the antenna array tank was filled with tap water, having dielectric properties *ϵ*_*r*_ = 77.5, *σ* = 0.05 S/m at 0.5 GHz. We also present a comparison between measured data and corresponding computed reflection and transmission coefficients. By replacing the air in the tank with water we further approach biologically relevant imaging scenarios. With the aim to study how modeling errors affect the reconstructed images we first studied how the forward modeling of the antenna system was affected by different antenna modeling errors.

#### 4.2.1. Full 3D Model

**Figure 4:**
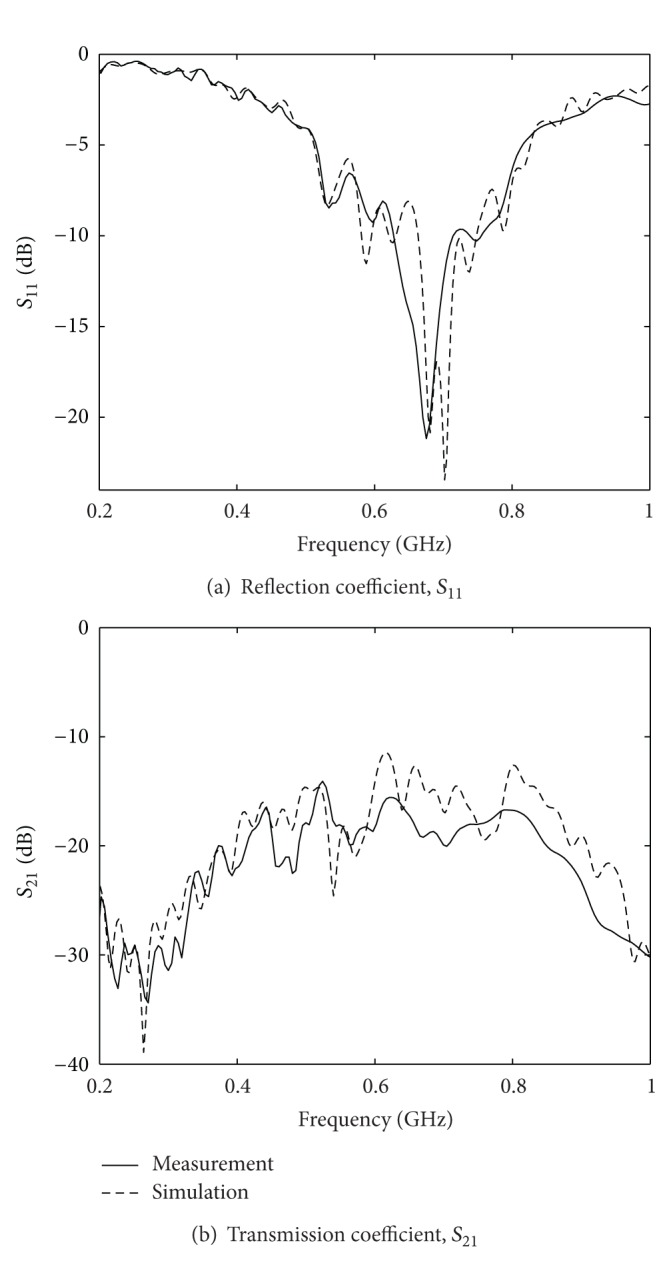
Comparison between measured and simulated reflection (*S*_11_) and transmission (*S*_21_) coefficients over the frequency range of interest for the imaging examples.

The experimental situation was such that the tank was filled with ordinary tap water up to a level of 50 mm above the ground plane. In the FDTD model measured dielectric values of the water at 0.5 GHz were used as this was in the center of the frequency spectrum used for the imaging. In the FDTD modeling care was also taken to represent the physical reality as accurately as possible, and the corresponding settings are summarized in Table 2. Modeling errors were introduced by varying some of the parameters in this table. Unfortunately it is not viable to show scattering data for all antenna combinations, but instead only a few representative cases are shown. Measured and simulated reflection and transmission coefficients between two adjacent antennas using the model parameters from Table 2 are shown in Figure 4. As can be seen, the agreement between the calculated and the measured data is very close. The calculated resonant frequency is 0.70 GHz compared with the measured resonant frequency of 0.67 GHz. There are ripples with approximately the same magnitude both in calculated and measured data and where some ripples agree with each other and some do not. The details about these ripples are further discussed in the following sections. The measured transmission coefficients are on average below −15 dB compared to the calculated transmission coefficient below −12 dB.

#### 4.2.2. Hard Source Feed Model

**Figure 5:**
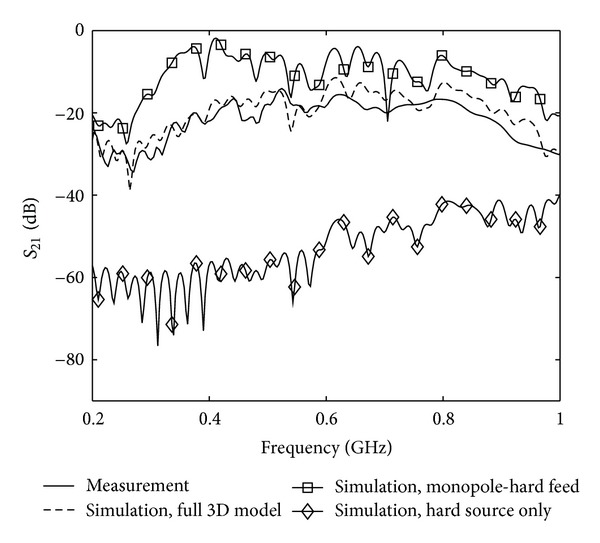
Measurement and simulations of the transmission coefficients *S*_21_ between two adjacent antennas. For ease of comparison the transmission data from Figure 4 has been kept and plotted together with the case when (1) The RVS has been replaced by a hard source and (2) the wire model has been removed and only a hard source has been used as transmitter.

Due to its simplicity, the hard source model is very appealing to use in FDTD simulations. However, it is not as accurate as the RVS when modeling the monopole feed. To quantify the errors associated with a hard source we investigate its applicability to model the monopole feed. In the first example, we replaced the RVS feed model with a hard source. In the second example, we used the hard source and completely removed the modeling of the rest of the monopole. The use of a hard source also implied that we could not use the transmitting antenna in the gradient computation as we do not calculate the reflection coefficient with the hard source model. Calculation of the reflection coefficient requires knowledge of the reflected wave, but since the E-field is directly set in the source cell, no update of the field will be made due to reflected waves. Instead we used only transmission data for the following reconstructions. As an illustration of the impact of the hard source on the simulated scattering data *S*_21_ for adjacent antennas has been plotted in Figure 5. For convenience the same measured and simulated data for the full 3D model as in Figure 4 has also been replotted in the same graph. As can be seen, in the first example, the RVS feed model improves the transmission coefficient data over the hard source model, and, in the second example, the system becomes very lossy due to the inaccurate model, and therefore the deviation from the measured data increases.

#### 4.2.3. Open Water Model

**Figure 6:**
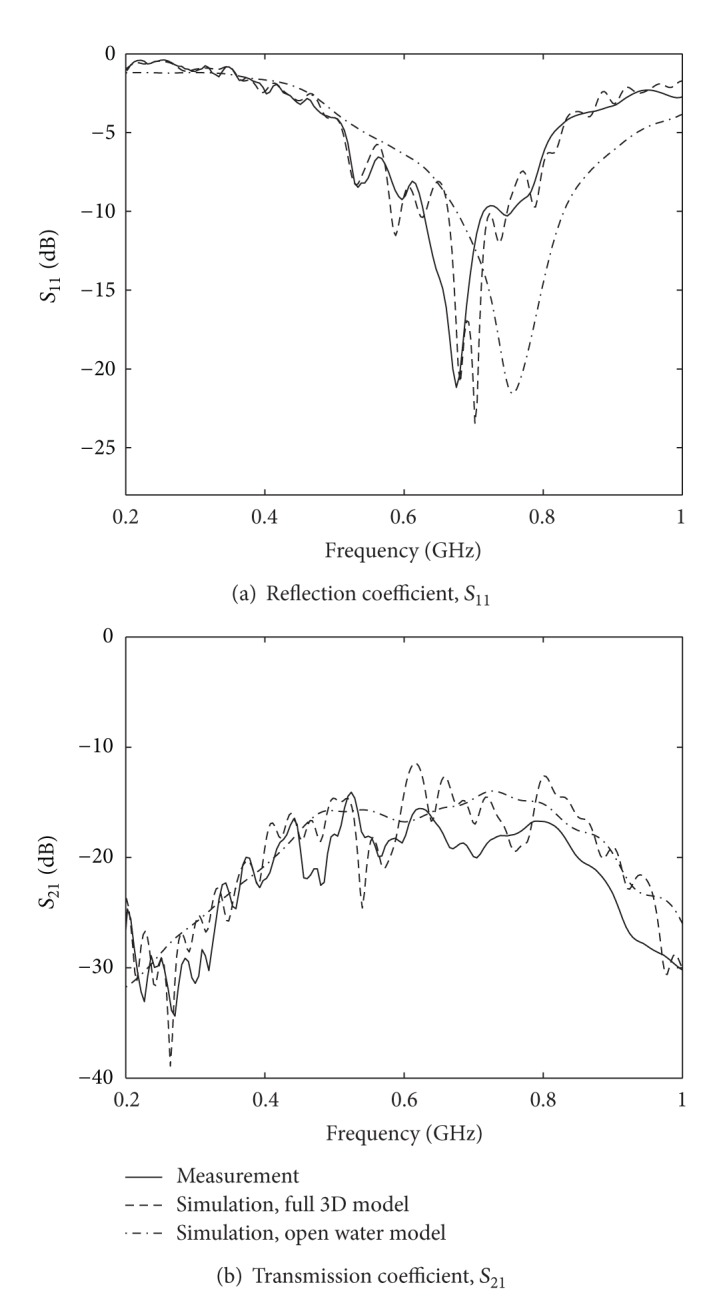
(a) Reflection coefficients with the antennas modeled in an infinite water background. For comparison the original measured and simulated data is shown. (b) Corresponding transmission coefficients for two adjacent antennas.

Solving the reconstruction problem is a computationally very demanding problem, and one strategy to reduce the simulation time is to reduce the size of the computational domain. Therefore we have investigated the need for modeling the exact volume of the water in the tank and instead modeled the water as a background directly terminated by CPML. The CPML is absorbing any outgoing wave and the result inside the computation domain is the same as if the simulation was made in an infinitely large space. In this FDTD simulation the entire computational grid was therefore assigned the properties of water, and as there were no need to model the water-air interface in the computational domain, the CPML could be moved closer to the antennas and thus the computational domain reduced. In Figure 6 the corresponding reflection, *S*_11_, and transmission, *S*_21_, coefficients have been plotted. For comparison the figures also contain the measured data and the data simulated with the full 3D FDTD model from Figure 4. The *S*_11_ data show how the computed resonance frequency has increased about 0.5 GHz, and we can also see how the fast varying ripple due to the water-air interface reflections has vanished. Similar changes can also be seen in the *S*_21_ data.

#### 4.2.4. 2D Model

**Figure 7:**
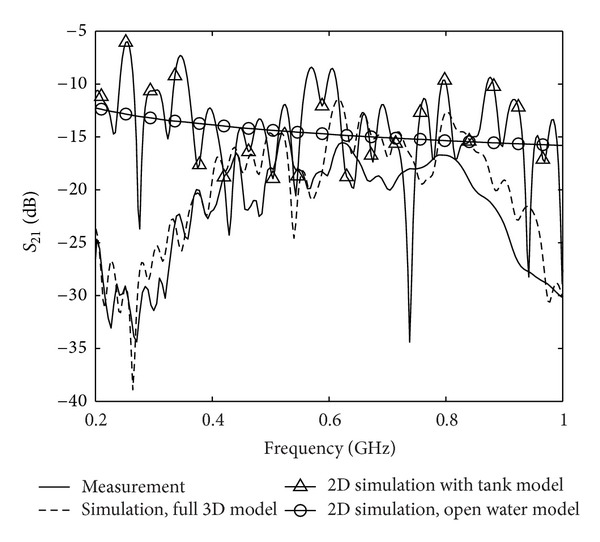
Transmission coefficients for the 2D FDTD model with the tank model and with the homogeneous background of water. For ease of comparison both the measured data and the simulation with the full 3D model are also shown.

We have also made a comparison with a 2D computational model. The reason is that the computation time is significantly reduced compared to the 3D case. Therefore a good understanding is desired of when the 2D approximation is applicable for imaging. In this section we show computed transmission data obtained with a 2D model, and also here two examples are considered. The first is the modeling of the volume of water in the tank as a 350 × 350 mm square in the 2D FDTD grid. In Table 3 the corresponding FDTD model parameters are summarized. In analogy with the 3D open water background model the second example was a 2D homogeneous background of water terminated with CPML. In Figure 7 transmission data is plotted for these two cases and again the measured data and the full 3D model data are shown. For the simulation data with the 2D square tank model the deviation from the measured data is of similar magnitude as for the case as with the hard source feed in Figure 5, at least around the center frequency 0.5 GHz. However, the amplitude of the ripple is larger and the deviation is much more significant. The fast varying ripple is clearly seen and is primarily due to the reflections from the water-air interface in the model. For the simulation in the homogeneous background this ripple is vanished, but in this case the computed data do not show much resemblance at all to the measured data.

### 4.3. Reconstruction of Targets Immersed in Water

In the following section, we show the reconstructed images from experimental scattering data. Two different target models were used and reconstructed using the various antenna array models discussed above. The aim was to investigate the impact on the reconstruction caused by the different modeling introduced in Section 4.2.

We have performed image reconstruction of targets immersed into ordinary tap water. In comparison to air a FDTD simulation of water is more time consuming due to the higher permittivity and the corresponding need for shorter time stepping in the FDTD algorithm. To save some computation time the reconstructions were therefore made using a multigrid technique where 10 iterations where performed on a (90 × 90 × 16) computational domain with grid size length 4 mm. The final reconstruction of the 10′th iteration was then taken as a starting point for 10 additional iterations on a (179 × 179 × 31) domain with grid size length 2 mm. The electromagnetic pulse had center frequency 0.5 GHz and FWHM bandwidth 0.5 GHz. The same pseudo-3D approach as previously described was used in a reconstruction domain of height 50 mm and radius *R*_*rd*⁡_ = 80 mm. Two different targets were used in the reconstructions. The first was made of a mixture of deionized water and ethanol resulting in permittivity *ϵ*_*r*_ = 57 and conductivity *σ* = 0.11 S/m at the frequency 0.5 GHz. The mixture was filled up to height 50 mm in a thin-walled plastic cup with diameter 42 mm and immersed into the water for the measurement. The second target was a plastic rod with permittivity *ϵ*_*r*_ ≈ 2.5, conductivity *σ* ≈ 0 S/m, and diameter 15 mm.

#### 4.3.1. Full 3D Model

**Figure 8:**
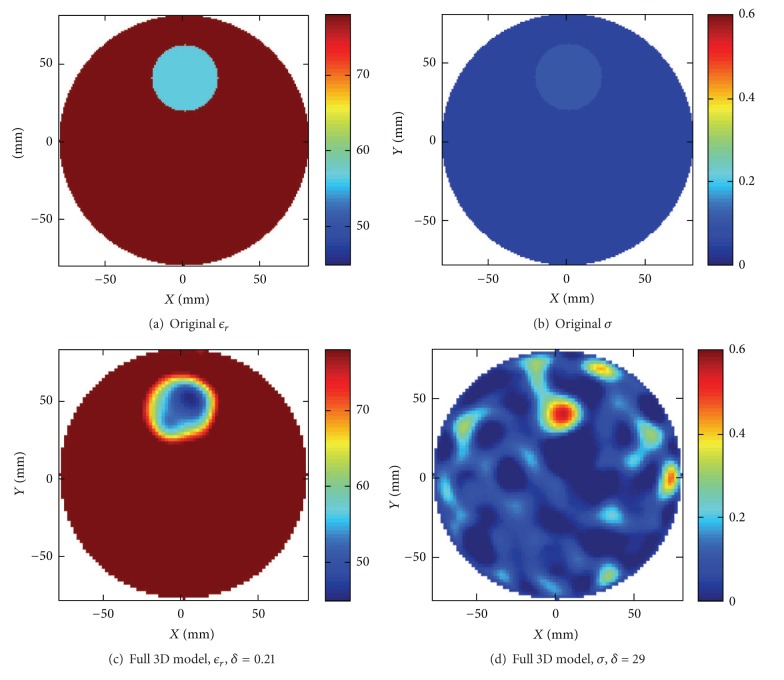
(a and b) Original configuration of the water/ethanol target immersed in water. (c and d) Reconstructions using the 3D algorithm.

Two different scenarios were reconstructed, the first with the center of the water/ethanol target positioned 39 mm from the center point of the antenna array. In Figure 8 an illustration of the original target is shown together with the reconstructed images. 

**Figure 9:**
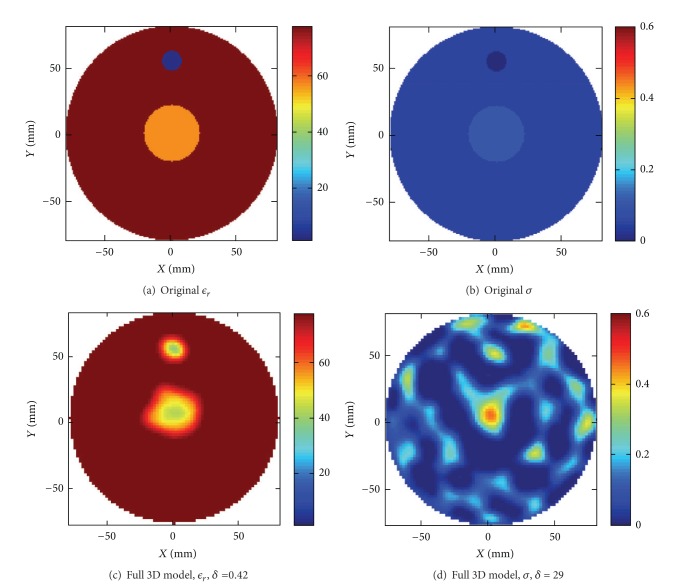
(a and b) Original configuration of the water/ethanol and plastic targets immersed in water. (c and d) Reconstructions using the 3D algorithm.

The second scenario that was reconstructed included both targets, the water/ethanol mixture and the plastic target, and these results are shown in Figure 9. In both cases we see that in the permittivity both size and position of the targets have been accurately reconstructed. The relative error for this second example with the two targets was computed to be *δ* = 0.42. In the conductivity we can perhaps see an indication of a target in the appropriate positions, but the image region is also cluttered with other artifacts, and comparison with the relative error is not meaningful. Furthermore the absolute values of the permittivity are not in total agreement with the original values, especially not for the small plastic target. With the same arguments as for the previous object in air where the conductivity also was badly reconstructed, it is not to expect anything else in this case. The difference between the background and the target made of water/ethanol mixture is about *ℜ*{Δ*ϵ*_r_*} = 20 in the real part but for the imaginary part only *ℑ*{Δ*ϵ*_r_*} = 2. For the plastic rod the difference is *ℜ*{Δ*ϵ*_r_*} = 75 and *ℑ*{Δ*ϵ*_r_*} = 2.

#### 4.3.2. Hard Source Feed Model

To assess the question of how the reconstructed image is affected by inaccuracies in the forward modeling we have taken the example with the two targets, the plastic rod, and the water/ethanol mixture and performed reconstructions with distorted electromagnetic models.

**Figure 10:**
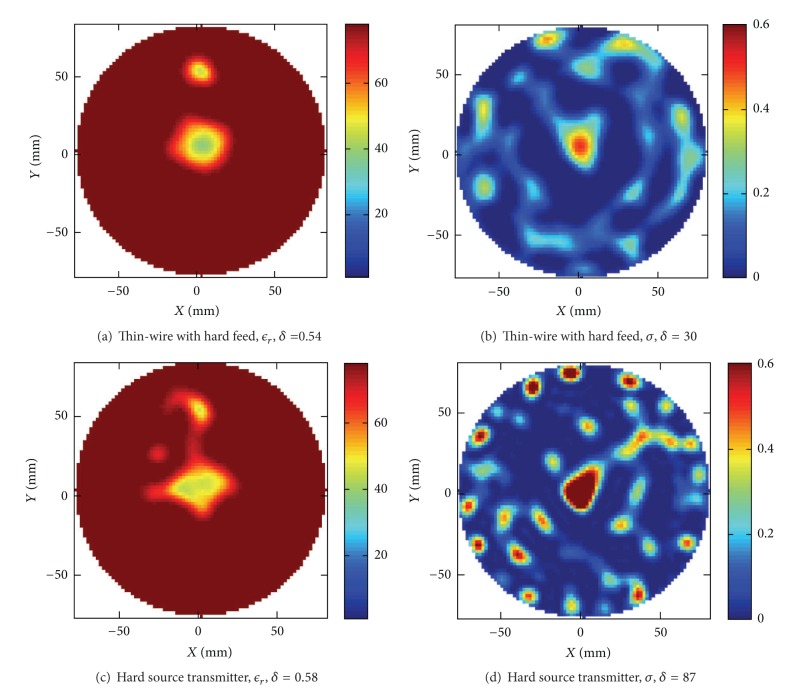
Reconstructions of the two target model from Figures 9(a) and 9(b). Here (a) and (b) show the reconstructed result when replacing the RVS feed of the monopole with a hard source. In (c) and (d) the results when removing the wire model of the antenna and using only a hard source as the transmitter model, and on the receiver side the field was directly sampled in the corresponding grid point.

Using the two altered antenna models with hard source feed new images of the original targets from Figures 9(a) and 9(b) have been reconstructed and shown in Figure 10. In (a) and (b) in this figure the RVS was replaced with the hard source feed, and in (c) and (d) the model of the monopole wire was completely removed and only the hard source was used as transmitter model. The relative errors in the permittivity images were computed to be *δ* = 0.54 and *δ* = 0.58, respectively. One can clearly see that, for the case where only the hard source was used to model the transmitter, the distortions of the reconstructed images are also the largest. This also corresponds to the case where the *S*-parameter data deviate the most from the measured data in Figure 5. However one should not believe that there exists a simple relation between the deviation between the measured and simulated data on one hand and the errors in the reconstructed images on the other hand. It is instead a highly nonlinear relation and a balance between the measurement, the reconstruction algorithm, the regularization, the modeling accuracy, and the calibration procedure. To summarize, so far the results shown indicate that the accuracy of the reconstructed image is highly influenced by the details in the antenna models and in the propagation model. So far we have also modeled the volume of water according to measurements of the level in the tank.

#### 4.3.3. Open Water Model

**Figure 11:**
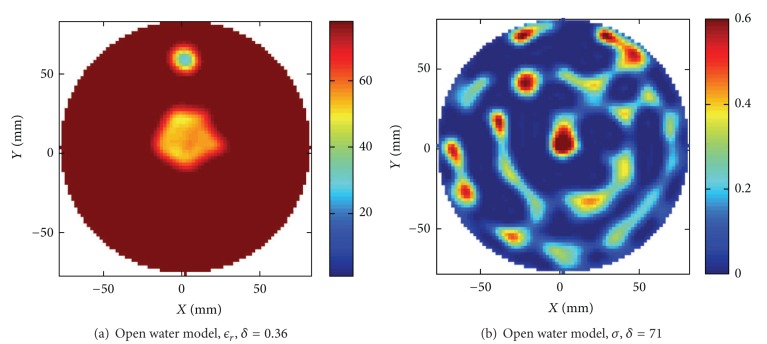
For an open space of water (a) and (b) show the dielectric distribution reconstructed with the 3D model.

We have investigated the need of actually modeling the exact extent of the water volume in the tank. For this experiment we used the RVS feed thin-wire monopole model, and the algorithmic settings were otherwise identical to the previous examples but with the dielectric properties of water assigned to all the grid cells in the computational domain which was then terminated by seven layers of CPML. The reconstruction can be seen in Figure 11. Interestingly the results are improved over the reconstructions made with the tank model, and, for example, the absolute values of the objects are better estimated with the 3D algorithm. The improvement is confirmed by the relative error which was calculated to be *δ* = 0.36 for the permittivity image, a decrease of about 14% compared to the reconstruction with the full 3D model in Figure 9. These results might be a bit surprising and contradictory to the idea that the better the forward model the better the reconstruction. However, we do not believe that it is the case. If we examine the scattering data in Figure 6 carefully, we see that even if the data simulated with the numerical tank model contains a similar ripple of the same magnitude as the measured data, the agreement in the details is not perfect. On average one can however approximately estimate the modeling errors in the two cases to be of similar magnitude in comparison to the measured data. Even if we have carefully created the model, the numerical grid is ultimately limiting the resolution and causing simulation errors. A refined grid should improve the modeling accuracy but that would also imply that the problem size increases beyond what can be practically handled by our computer cluster due to memory and simulation time requirements. These results suggest that we in this situation are better off by using the simpler open water model and relying on the calibration to resolve the remaining discrepancy between the simulated and the measured data. This is not a result that is obvious to predict but instead a result of the nonlinear property of the problem. In practice the optimal numerical model would thus have to be determined from case to case in different imaging situations and with different imaging systems.

#### 4.3.4. 2D Model

**Figure 12:**
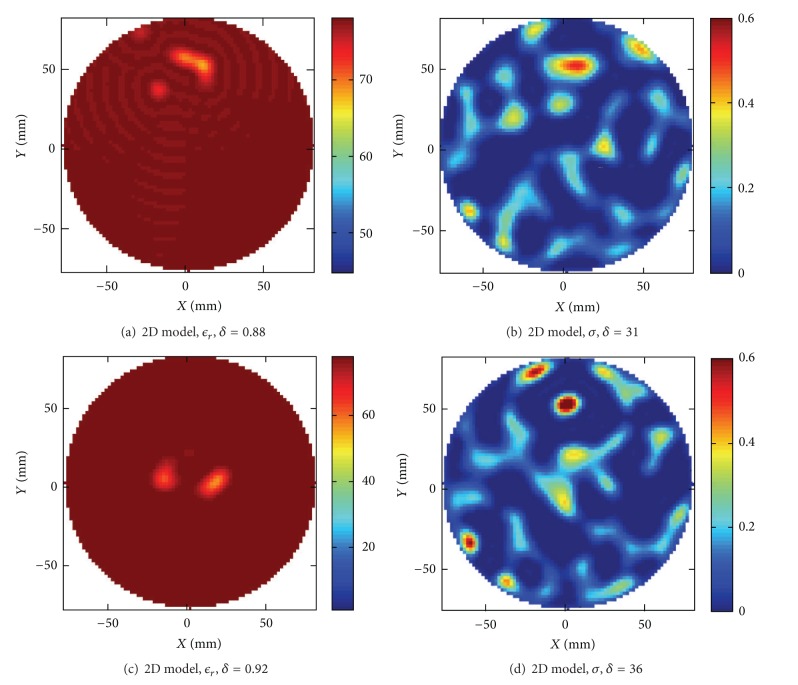
Reconstructed images using the 2D model. The water tank was modeled as a square in a background medium consisting of air. (a) and (b). Reconstruction of the original target from Figures 8(a), 8(b), 8(c), and 8(d). Reconstruction of the original target from Figures 9(a) and 9(b).

**Figure 13:**
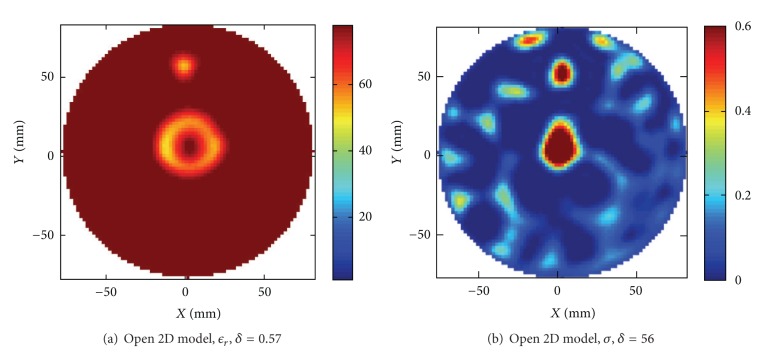
Reconstructed images using the open water 2D model. The water tank was modeled as a homogeneous background of water where the computation domain was terminated by CPML to model an open domain. (a) and (b) Reconstruction of the original target from Figures 9(a) and 9(b).

For comparison we have also performed image reconstruction of the same targets using a 2D version of the algorithm. The first reconstruction made with the water tank modeled as a square of the single target as shown in Figures 8(a) and 8(b), and the reconstructed images are shown in Figures 12(a) and 12(b). The corresponding reconstruction of the scenario with two objects as shown in Figures 9(a) and 9(b) is shown in Figures 12(c) and 12(d). In these reconstructions we hardly see any object at all. Previous reconstructions made in air of sunflower oil targets with the 2D algorithm published in [12] showed a serious distortion of the size and dielectric values of the targets but a qualitatively correct image could usually be obtained. The reason for the distortion of the reconstructed objects could be attributed to 2D approximation errors as the targets only had the same height as the monopoles. It is a bit surprising that the reconstructed images shown in this paper of targets immersed in water hardly shows anything at all as the electromagnetic waves now in fact should be better confined inside the layer constituted by the water and therefore better conforming to the 2D approximation. Enclosure is provided by the ground plane on the bottom and the impedance step in the water-air interface. However these images are representative for our results showing that robust image reconstruction is not possible to achieve in water using this 2D algorithm. The situation can be somewhat improved by employing the open water modeling also in 2D; that is, we replace the square water tank model in the background of air with a modeling of a homogeneous background of water to simulate an open domain. The corresponding reconstruction is plotted in Figure 13. In this reconstruction we clearly see the two objects appearing, where the large object contains a spurious hole and the dielectric values are not quite close to the original values. Even if the situation is improved, this reconstruction cannot be considered satisfactory. A similar spurious hole in the reconstruction can be seen also in the reconstructions from short monopole lengths in Figure 2 and arises due to the modeling errors of the antenna. Furthermore associated with every target object is an optimal spectral content that will produce an optimal image [17], and holes usually appear when the spectral content is moved towards higher frequencies. With these two causes for the inaccuracy in the image we have not been able to improve the outcome any further by tuning the parameters in the reconstruction algorithm.

## 5. Conclusion

We have shown successful reconstruction of single and multiple targets immersed in water using a FDTD-based 3D reconstruction algorithm. By investigating various imaging scenarios we have also assessed the question of how the accuracy in the numerical forward model affects the reconstructed image. The results show that a realistic model of the antennas is necessary to achieve robust and reliable imaging. The same results also indicate that small modeling errors, such as in the length of the monopole, can have a clearly evident impact on the resulting image. For a simple antenna such as a monopole the modeling is very simple, and it is not difficult to produce a reasonably accurate numerical model using the thin-wire approximation and the RVS. However there is always an inherent limitation due to the finite grid size, and, when moving to more advanced antennas, such as patch antennas, modeling accuracy will become a more challenging task. Accurate antenna modeling requires a fine FDTD grid, but at the same time we must keep the grid as coarse as possible to lower the simulation time and memory requirement. Furthermore we have seen that when filling the tank containing the antenna system with normal tap water, the water-air interface causes significant reflections that are clearly identified in the scattering data. Despite this effect the accuracy of the reconstructed results is in fact improved when we instead use an open water model. This result shows that an apparent improvement in the antenna modeling and the corresponding computed scattering data is in fact not beneficial when it comes to imaging. One possible explanation could be that when comparing different models with similar modeling errors the nonlinearity of the reconstruction problem makes it impossible to predict the outcome. Instead the result will be strongly case dependent and to fully predict the result one would have to perform detailed studies of the particular imaging scenario.

## Figures and Tables

**Table 1:** Specifications of the 3D FDTD modeling and reconstruction parameters for the sun flower oil target in an otherwise empty antenna system.

FDTD grid	149 × 149 × 38
Grid size length	2 mm
CPML	7 layers
Pulse center frequency	2.3 GHz
Pulse FWHM bandwidth	2.3 GHz
Water level	No water in tank
Background properties	*ϵ*_*r*_ = 1.0, *σ* = 0.0 S/m
Antenna model	Thin wire
Feed model	50 *Ω* RVS

**Table 2:** Specifications of the 3D FDTD modeling when the antenna system was filled with water.

FDTD grid	179 × 179 × 35
Grid size length	2 mm
CPML	7 layers
Pulse center frequency	0.5 GHz
Pulse FWHM bandwidth	0.5 GHz
Water level in tank	50 mm
Water properties	*ϵ*_*r*_ = 77.5, *σ* = 0.05 S/m
Background properties	*ϵ*_*r*_ = 1.0, *σ* = 0.0 S/m
Antenna model	Thin wire
Feed model	50 *Ω* RVS

**Table 3:** Specifications of the 2D FDTD modeling when the antenna system was filled with water.

FDTD grid	179 × 179
Grid size length	2 mm
CPML	7 layers
Pulse center frequency	0.5 GHz
Pulse FWHM bandwidth	0.5 GHz
Water properties	*ϵ*_*r*_ = 77.5, *σ* = 0.05 S/m
Background properties	*ϵ*_*r*_ = 1.0, *σ* = 0.0 S/m
Transmitting antenna model	Hard source
Receiving antenna model	Field is sampled
